# Temperature Effect on the Adsorption and Volumetric Properties of Aqueous Solutions of Kolliphor^®^ELP

**DOI:** 10.3390/molecules25030743

**Published:** 2020-02-09

**Authors:** Katarzyna Szymczyk, Magdalena Szaniawska, Joanna Krawczyk

**Affiliations:** Department of Interfacial Phenomena, Institute of Chemical Sciences, Faculty of Chemistry, Maria Curie-Skłodowska University in Lublin, Maria Curie-Skłodowska Sq. 3, 20-031 Lublin, Poland; magdalena.szaniawska@poczta.umcs.lublin.pl (M.S.); j.krawczyk@poczta.umcs.lublin.pl (J.K.)

**Keywords:** Kolliphor^®^ ELP, volume expansivity, thermo-acoustic parameters, Gruneisen parameter, fractional free volume, coiling and micropolarity index

## Abstract

Density, viscosity and surface tension of Kolliphor^®^ ELP, the nonionic surfactant aqueous solutions were measured at temperature T = 293–318 K and at 5K interval. Steady-state fluorescence measurements have been also made using pyrene as a probe. On the basis of the obtained results, a number of thermodynamic, thermo-acoustic and anharmonic parameters of the studied surfactant have been evaluated and interpreted in terms of structural effects and solute–solvent interactions. The results suggest that the molecules of studied surfactant at concentrations higher than the critical micelle concentration act as structure makers of the water structure.

## 1. Introduction

Considering the landscape of current drug development, it can be stated, that40% of NCE (new chemical entities) are characterized by poor water solubility [1]. Therefore, there is a need for excipients to solubilize such candidates in both the early preclinical and clinical evaluation, as well as for the development of the marketed drug dosage forms. Solubilization is the process of drug uptake through complex formation into, e.g., oligomers of dextrose and fatty acids, through the cosolvent systems (such as ethanol, polyethyleneglycol and glycerol), or through the surfactant systems [2]. Contrary to the expectation that pharmaceutical excipients are pharmacologically inactive, there is abundant evidence that they can influence drug metabolism and efflux transport [3,4,5]. Some surfactants, particularly nonionic Kolliphors which were known earlier as Cremophors, find application in pharmaceutical formulations as the forms of solid dosage and delivery systems based on lipids aimed at improvement of poorly water-soluble drugs bioavailability [2,6]. The triricinoleate ester of ethoxylated glycerol is the main component of Kolliphors. The others are polyethylene glycol ricinolates, as well as the corresponding free glycols [7]. Kolliphor^®^ EL (EL), being the most often used Kolliphor, was applied as a vehicle in the case of solubilization of some hydrophobic drugs, including cyclosporin A, diazepam, propofol and paclitaxel [8]. However, some researchers stated that EL is not an inert vehicle but exerts a range of biological effects, some of which have important clinical implications. Its use was associated with severe anaphylactoid hypersensitivity reactions, hyperlipidemia, abnormal lipoprotein patterns, aggregation of erythrocytes and peripheral neuropathy. The pharmacokinetic behavior of EL is dose-independent, although its clearance is highly influenced by the duration of the infusion [9,10]. This is particularly important since EL can affect the disposition of various drugs by changing the unbound drug concentration through micellar encapsulation. From this point of view, a clear understanding of the biological and pharmacological role of surfactants is essential to help oncologists avoid side-effects associated with the use of paclitaxel or other agents. On the other hand to describe the behavior of surfactants in relation to two non-miscible phases and the range of temperatures at which they are active, some adsorption and volumetric properties of the solution of such surfactants are needed. Thus, the purpose of the presented studies was to determine some adsorption, volumetric, thermo-acoustic as well as anharmonic properties of aqueous solutions of Kolliphor^®^ ELP (ELP), a purified grade of EL, by surface tension, density and viscosity measurements at T = 293–318 K with 5 K interval. Moreover, the properties of the solutions were studied by means of steady-state fluorescence measurements. Based on the results, the analysis was applied to study different molecular interactions in the solutions taking into account the change of these properties depending on concentration and temperature.

## 2. Results and Discussion

**Figure 1:**
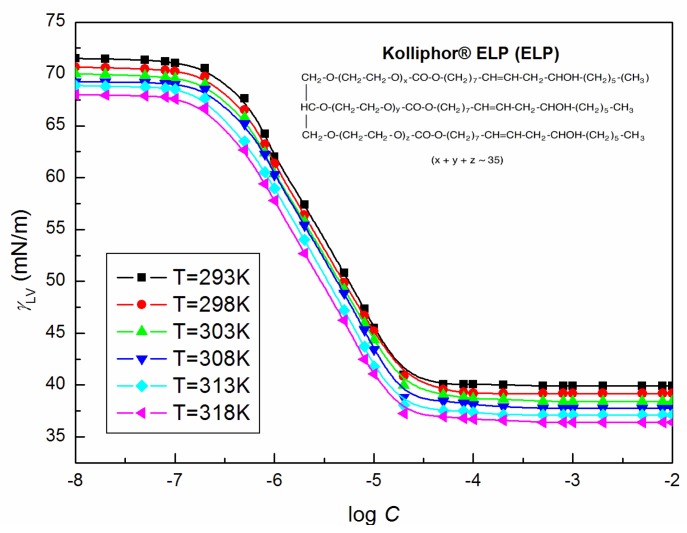
A plot of the values of the surface tension ($\gamma_{LV}$) of the aqueous solution of Kolliphor ^®^ ELP (ELP) at T = 293 K, 298 K, 303 K, 308 K, 313 K and 318 K vs. the logarithm of the surfactant concentration, log *C*.

Table 1 presents the calculated different parameters determining the surface and bulk properties of aqueous solutions of ELP based on the values of their surface tension ($\gamma_{LV}$) (Figure 1). These parameters include: *CMC* (critical micelle concentration), surfactant efficiency for water surface tension reduction, which means the concentration of the surfactant for 20 mN/m reduction of the surface tension ($C_{20}$), preference of the surfactant for adsorption in relation to the micelle formation with the pointer indicating possible reduction of water surface tension due to the surfactant presence ($CMC/C_{20}$), the surface pressure at the *CMC* ($\Pi_{CMC}$), the surface excess concentration at the surface saturation ($\Gamma_{m}$), the minimum surface area per molecule ($A_{m}$), as well as the standard free enthalpy ($\Delta G_{mic}^{0}$ and $\Delta G_{ad}^{0}$), enthalpy ($\Delta H_{mic}^{0}$ and $\Delta H_{ad}^{0}$) and entropy ($\Delta S_{mic}^{0}$ and $\Delta S_{ad}^{0}$) of micellization and adsorption [11,12,13]. For studied ELP solutions, $\Gamma_{m}$ and $A_{m}$ were calculated from the equations:

(1) $$\Gamma_{m}=-\frac{Cd\gamma_{LV}}{RTdC}=-\frac{1}{RT}\frac{d\gamma_{LV}}{d\ln C}=-\frac{1}{2.303RT}\frac{d\gamma_{LV}}{d\log C}$$

(2) $$A_{m}=\frac{1}{N\Gamma_{m}}$$

where *C* represents the concentration of surfactant, *R* is a gas constant, *T* is temperature and *N* is an Avogadro number [11,12]. Next, the values of $\Delta G_{mic}^{0}$ and $\Delta G_{ad}^{0}$ were determined:

(3) $$\Delta G_{mic}^{0}=RT\ln\frac{CMC}{\omega }$$

(4) $$\Delta G_{ad}^{0}=\Delta G_{mic}-\left(\frac{\Pi_{CMC}}{\Gamma_{m}}\right)$$

where ω is the number of water moles in 1 dm^3^.

Knowing the values of $\Delta G_{}^{o}$ at different temperatures, it was possible to calculate $\Delta H_{}^{o}$ and $\Delta S_{}^{o}$. If it is assumed that in a range of temperature, $\Delta H_{}^{o}$ is constant, then:

(5) $$\frac{d\left(\Delta G_{}^{0}\right)}{dT}=-\Delta S_{}^{0}$$

On the other hand, if $\Delta S_{}^{o}$ is constant, it is obtained [11]:

(6) $$T^{2}\frac{d\left(\frac{\Delta G_{}^{0}}{T}\right)}{dT}=-\Delta H_{}^{0}$$

As follows from the table, the calculated values of $\Delta G_{mic}^{0}$ and $\Delta G_{ad}^{0}$ are negative, indicating that the ELP molecules have a trend to adsorb at the water–air interface and to form micelles in the bulk phase, as well as the two processes are spontaneous. The adsorption free enthalpy values are more negative in comparison with the micellization values indicating greater propensity of ELP for adsorption at the interface than micelles formation in the bulk phase. The $\Delta G_{mic}^{0}$ and $\Delta G_{ad}^{0}$ values become more negative with the increasing temperature probable due to the greater stability of the adsorbed and micellized molecules of ELP compared to the freely dispersed in the aqueous phase. However, the positive values of $\Delta S_{ads}^{0}$ show that, after adsorption and micellization, the studied solutions become more random [11]. On the other hand, $\Delta H_{mic}^{0}$ and $\Delta H_{ads}^{0}$ positive values the indicate predominance of bond breaking when micellization and adsorption proceed. Figure 1 shows the gradual decrease of ELP surface tension values with the increasing temperature being 293–318 K, which is similar to the $\Gamma_{m}$,$\Pi_{CMC}$ and $CMC/C_{20}$ values (Table 1). As follows from the data presented in Table 1, the ELP CMC values drop slightly with the temperature rise from 293 to 318 K. This indicates that temperature increase can break down the intra-hydrogen bonds between the surfactant molecules and weaken the hydration action of the hydrophilic groups, which favors the micelle formation [14].

**Figure 2:**
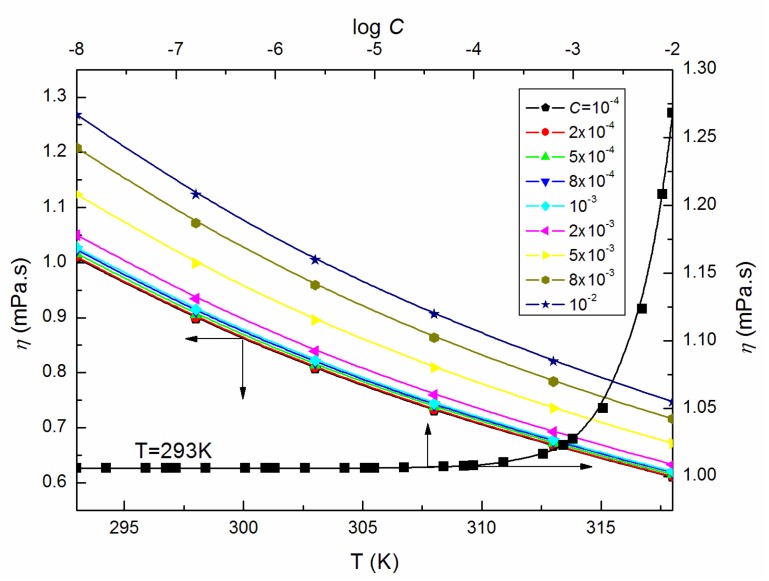
A plot of the values of η of aqueous solutions of ELP at *C* from 10^−4^ to 10^−2^ M vs. the temperature, T, as well as the values of η of the aqueous solutions of ELP at T = 293 K vs. log *C*.

The surface tension isotherm ($\gamma_{LV}$ = f (log *C*)) is known to not be the only way to determine *CMC.* What is more, the values of *CMC* for a surface-active agent very often differ depending on its determination method [15]. Considering the values of the dynamic viscosity (η) of ELP aqueous solutions (Figure 2 as an example), it is evident that the great increase of the η values take place at 293 K and the concentration exceeding 10^−3^ M. 

**Figure 3:**
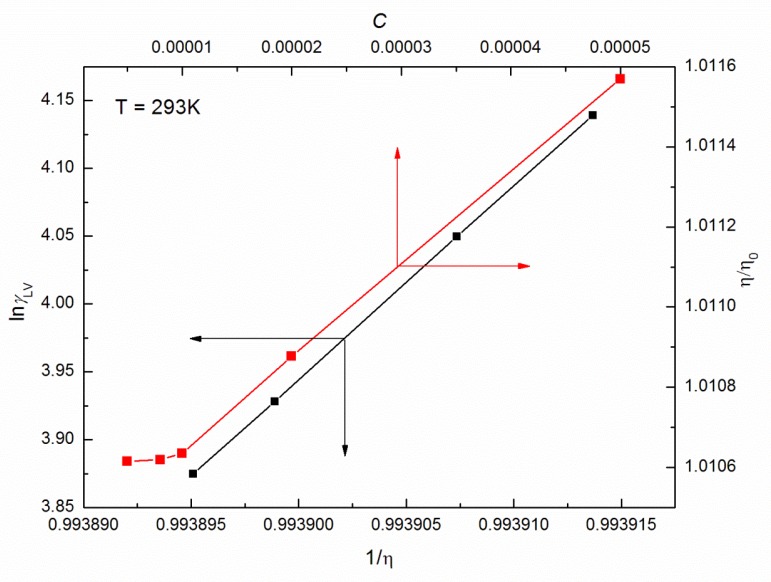
A plot of the values of the $\ln\gamma_{LV}$ vs. 1/η as well as $\eta /\eta_{0}$ vs. *C* for the aqueous solutions of ELP at T = 293 K.

Based on the relations between the relative viscosity ($\eta /\eta_{0}$) and concentration *C* [16] of ELP at a given T (Figure 3 as an example), the *CMC* values are as follows: 9.87 × 10^−6^ M, 9.84 × 10^−6^ M, 9.73 × 10^−6^ M, 9.66 × 10^-6^ M, 9.52 × 10^−6^ M and 9.32 × 10^−6^ M in the temperature range 293–318 K. They are similar to the values given in Table 1. However, besides the determination of *CMC* from the viscosity measurements, it was attempted to relate the surface tension with viscosity [17]. Moreover, the basic equation proposed by Pelofsky is the linear relation and can be used for organic and inorganic phases of pure and mixed components [18]. It is interesting that, taking into account the measured values of $\gamma_{LV}$ and η at a given temperature, and *C* corresponding to the saturated monolayer at the water–air interface, there is the linear dependence proposed by Pelofsky that is between $\ln\gamma_{LV}$ and 1/η for the ELP solutions (Figure 3). 

**Figure 4:**
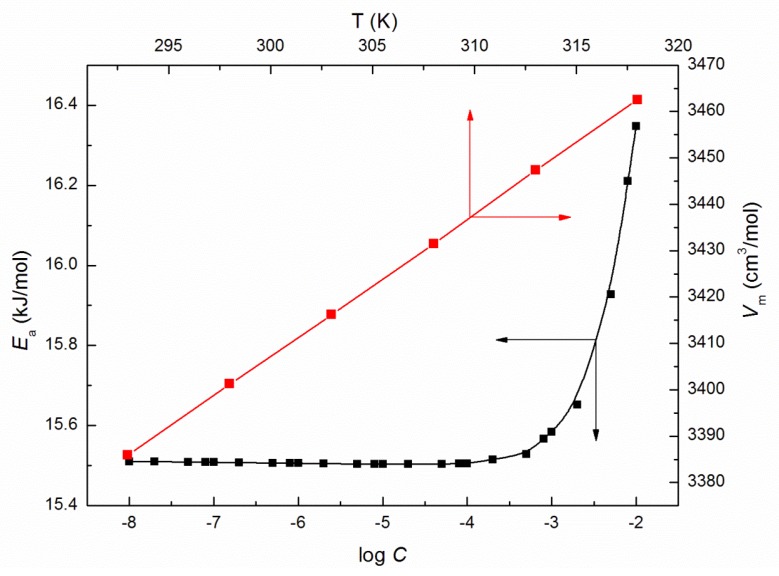
A plot of the values of shear activation energy ($E_{a}$) of the aqueous solutions of ELP vs. log *C*, as well as the values of the partial molar volume ($V_{M}$) of the aqueous solutions of ELP vs. the temperature, T.

**Figure 5:**
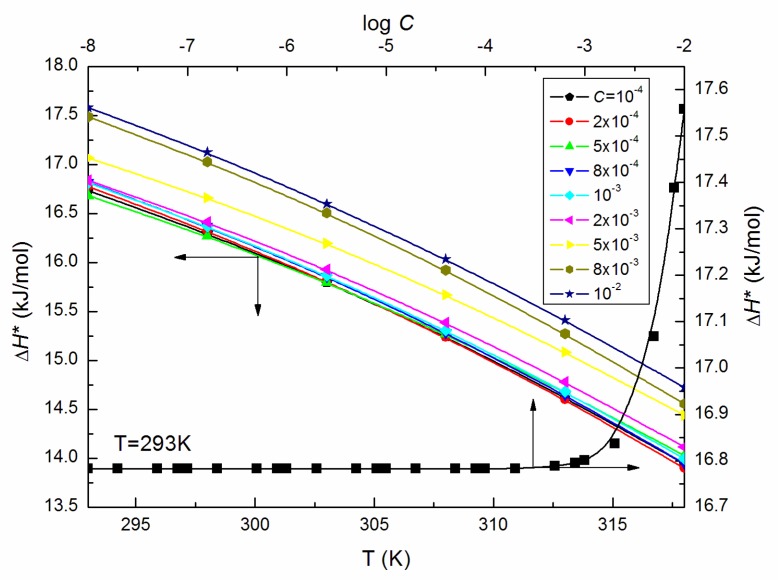
A plot of the values of the enthalpy of activation ($\Delta H^{*}$) of the aqueous solutions of ELP at *C* from 10^−4^ to 10^−2^ M vs. the temperature, T, as well as the values of $\Delta H^{*}$ of the aqueous solutions of ELP at T = 293 K vs. log *C*.

**Figure 6:**
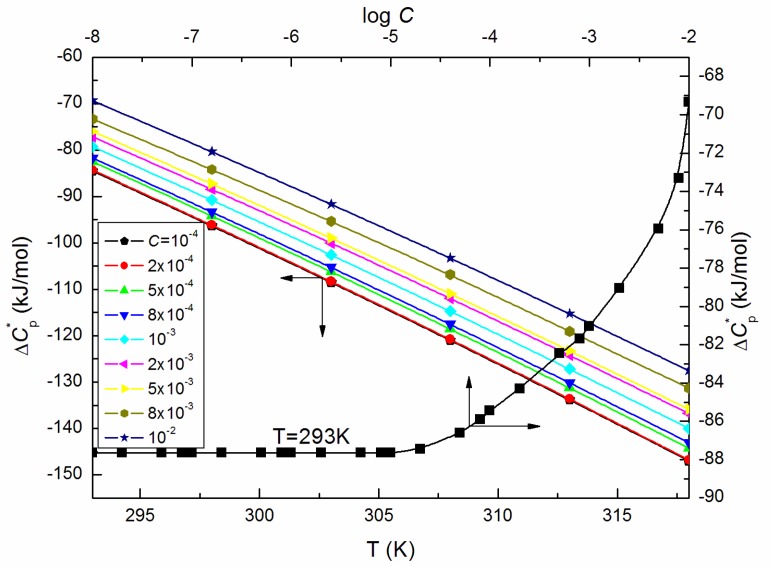
A plot of the values of the change in heat capacity of activation ($\Delta C_{p}^{*}$) of the aqueous solutions of ELP at *C* from 10^−4^ to 10^−2^ M vs. the temperature, T, as well as the values of $\Delta C_{p}^{*}$ of the aqueous solutions of ELP at T = 293 K vs. log *C*.

On the other hand, the constant *B* in the relation presented by the author as a function of the molecular weight and thermal conductivity possesses a positive value at each temperature contrary to the values for *n*-alkanes, *n*-alcohols, water as well as some aqueous solutions [17]. In our opinion, the *B* parameter values can be better explained by conducting additional measurements for a larger amount of surfactant concentrations. Therefore, it is certain that the measured dynamic viscosity (η) of ELP solutions is greatly sensitive to changes in temperature (exemplary Figure 2) following the Arrhenius law [19,20]:

(7) $$\eta =B\exp\frac{E_{a}}{RT}$$

where *B* is the is pre-exponential factor and $E_{a}$ is shear activation energy. The calculated from Equation (7) values of $E_{a}$ (Figure 4), that is energy necessary for individual micelles motion in an environment of surrounding micelles determined on the basis of this law, increases significantly at ELP concentrations in the bulk phase higher than 10^−3^ M. Moreover, the highest value of $E_{a}$ being 16.35 kJ/mol is found at *C* = 10^−2^ M. Furthermore, the enthalpy of activation ($\Delta H^{*}$) values, as well as the change in heat capacity of activation ($\Delta C_{p}^{*}$), are affected to a great extent by temperature (exemplary Figure 5 and Figure 6). The values of $\Delta H^{*}$ and $\Delta C_{p}^{*}$ were determined based on the relations proposed by Mukherjee et al. [21,22]:

(8) $$-\frac{\Delta H^{*}}{RT^{2}}=\frac{d\ln\eta }{dT}$$

(9) $$\Delta C_{p}^{*}=\frac{d\Delta H^{*}}{dT}$$

What is more, the $\Delta H^{*}$ values calculated from Equation (8) for the ELP solutions are positive and expectedly declined with increasing temperature (Figure 5) indicating that the processes are connected with the heat absorption in the solutions under considerations. From this reason, the $\Delta C_{p}^{*}$ values are negative and decrease linearly with *T* (Figure 6).

**Figure 7:**
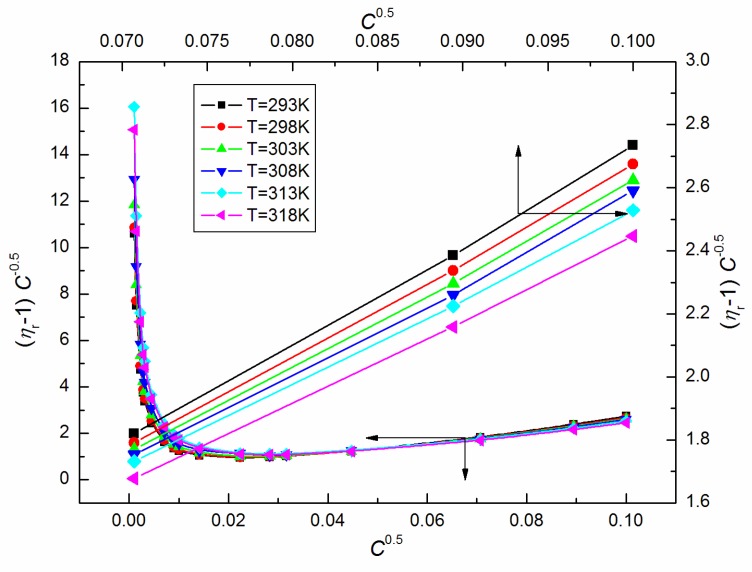
A plot of the values of $\left(\eta_{r}-1\right)C^{-0.5}$ of the aqueous solutions of ELP at T = 293 K, 298 K, 303 K, 308 K, 313 K and 318 K vs. $C^{0.5}$

**Figure 8:**
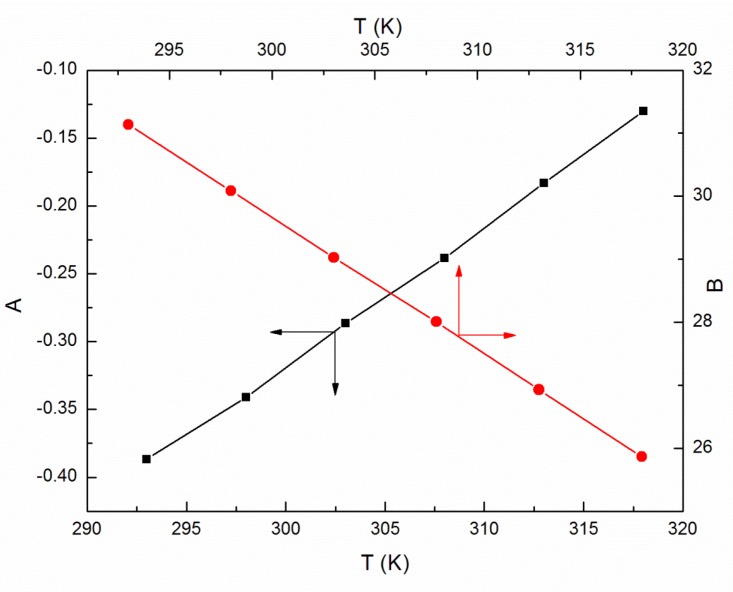
A plot of the values of A and B coefficients determined from the Jones–Dole equation and viscosity of the aqueous solutions of ELP vs. T.

**Figure 9:**
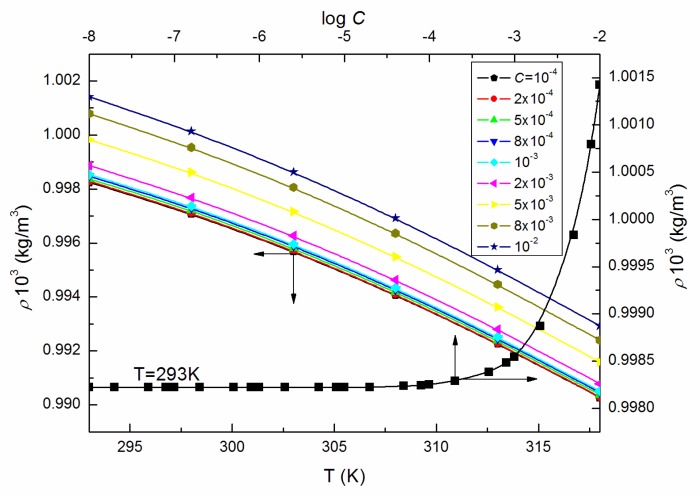
A plot of the values of ρ of the aqueous solutions of ELP at *C* from 10^−4^ to 10^−2^ M vs. the temperature, T, as well as the values of ρ of the aqueous solutions of ELP at T = 293 K vs. log *C*.

**Figure 10:**
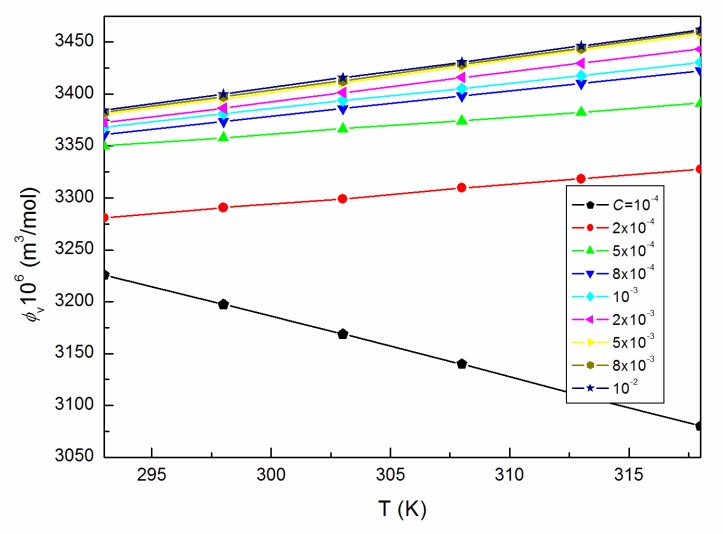
A plot of the values of the apparent molar volume, $\varphi_{V}$, of the aqueous solutions of ELP at *C* from 10^−4^ to 10^−2^ M vs. the temperature, T.

As the ELP molecules are strongly hydrated by water, and it is possible that the H_3_O^+^ ions can be associated to the oxyethylene groups, similarly to other nonionic surfactants studied by us [15,22,23,24,25], it was also interesting to analyze the viscosity data according to the Jones–Dole equation and A and B coefficients [26,27]. This equation and the relationship between $\left(\eta_{r}-1\right)C^{-0.5}$ and $C^{0.5}$ (Figure 7) for *C* equal to and higher than 5 × 10^−3^ M, where $\eta_{r}$ is the relative viscosity, were used for the determination of B coefficients values which proved to be positive (Figure 8), disclosing the water-structure-breaking nature of the ELP molecules. It is interesting that the value of dB/dT, which by some authors is designed as a better criterion for determining any solute effect on the structure of solutions depending on temperature [28], for the studied concentrations of ELP solutions, is equal to −0.21, and according to Hugue et al. [28], indicates that the solute is a structure maker. To solve an idea about the structure-making or breaking role of the ELP in the solution, the apparent molar volume values, $\varphi_{V}$ [29,30], were determined from the density measurements (ρ) (exemplary Figure 9 and Figure 10) and the equation which has the form:

(10) $$\varphi_{V}=\frac{M}{\rho_{0}}+\frac{1000\left(\rho_{0}-\rho \right)}{C}$$

where M is the molecular weight of the surface-active agent and $\rho_{0}$ is the density of the “pure” solvent. The calculated values of $\varphi_{V}$ were then analyzed based on the Hepler equation [31]. It proved that at *C* equal to and greater than 5 × 10^−3^ M, the values of ${\left(\partial^{2}\varphi_{{}_{V}}^{0}/\partial T^{2}\right)}_{p}$, where $\varphi_{{}_{V}}^{0}$ is the apparent molar volume at infinite dilution, are positive, indicating the structure-making properties of the investigated surfactant.

**Figure 11:**
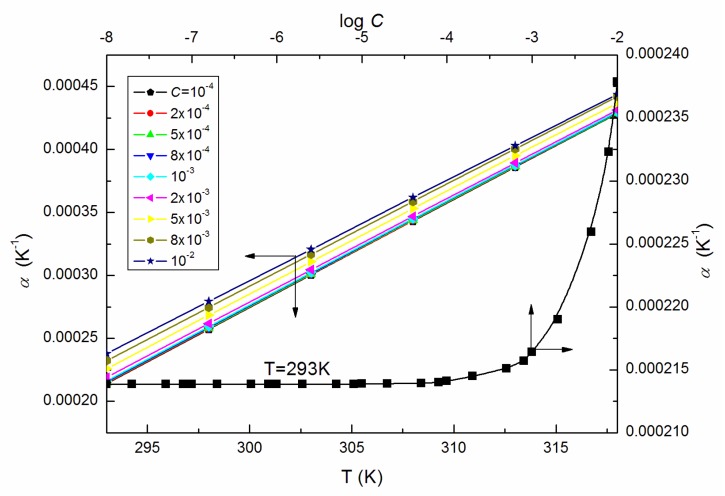
A plot of the volume expansivity α of the aqueous solutions of ELP at *C* from 10^−4^ to 10^−2^ M vs. the temperature, T, as well as the values of α of the aqueous solutions of ELP at T = 293 K vs. log *C*.

However, the changes of the $\varphi_{V}$ values with *T* before and after *C* = 10^−4^ M (shown in Figure 10) are different pointing out the great structural changes of ELP molecules due to the increase in temperature and/or those of intermolecular interactions between the surfactant and water. They, in turn, can be determined, among others, based on the volume expansivity values α (Figure 11), which are also regarded as the thermal expansion coefficient being a measure of volume change with the temperature and can be calculated based on the measurements of density from the equation [18,28]:

(11) $$\alpha =\frac{1}{V_{m}}\left(\frac{dV_{m}}{dT}\right)$$

where $V_{m}$ is the partial molar volume. It follows from Figure 11 that for ELP, the calculated values of α increase with the rise of *T* and *C*, which is consistent with the increasing tendency of $V_{M}$ for studied solutions (Figure 4) at the concentrations higher than *CMC* of the studied surfactant determined from the surface tension measurements (Table 1). 

Having the ELP α values at various temperatures and concentrations, there could be calculated the thermodynamic parameters such as: reduced volume ($\tilde{V}$), Moelwyn–Hughes parameter ($C_{1}$), reduced compressibility ($\tilde{\beta }$), isochoric temperature coefficient of internal pressure (X), Sharma parameter ($S_{0}$), Huggin’s parameter (F), isochoric temperature coefficient of volume expansivity ($X^{\prime }$), anharmonic microscopic isothermal Gruneisen parameter (Γ), fractional free volume (f), Gruneinsen parameter ($\Gamma_{p}$), isobaric thermo-acoustic parameter (K) and isochoric thermo-acoustic parameter ($K^{\prime \prime }$) [32,33,34,35]. These parameters for ELP solutions are presented in Table S1 (Supplementary Materials) and were calculated from the following equations:

(12) $$\tilde{V}={\left\{\frac{\left(\frac{\alpha T}{3}\right)}{1+\alpha T}+1\right\}}^{3}$$

(13) $$C_{1}=\left(\frac{13}{3}\right)+\left(\frac{1}{\alpha T}\right)+\left(\frac{4\alpha T}{3}\right)$$

(14) $$\tilde{\beta }={\left[{\tilde{V}}^{C_{1}}\right]}^{-1}$$

(15) $$X=-\frac{2\left(1+2\alpha T\right)}{{\tilde{V}}^{C_{1}}}$$

(16) $$S_{0}=\left(-\frac{X}{2}\right)\left(3+4\alpha T\right)$$

(17) $$F=2\left[1+\left(\frac{S_{0}}{3+4\alpha T}\right)\right]-\left(3+\frac{4\alpha T}{3}\right)$$

(18) $$X^{\prime }=-\left(1+2\alpha T\right)$$

(19) $$\Gamma =\left(\frac{2}{3}\right)\alpha T+\left(\frac{2-F+4\alpha T}{2\alpha T}\right)$$

(20) $$f=\frac{1}{\left(\Gamma +1\right)}$$

(21) $$\Gamma_{p}=\left(\frac{2}{3}\right)\alpha T+\left(\frac{1}{2\alpha T}\right)+2$$

(22) $$K=\frac{1}{2}\left[1+\frac{\left(1+\frac{4\alpha T}{3}\right)\left(1+\alpha T\right)}{\alpha T}\right]$$

(23) $$K^{\prime }=\frac{1}{2}\left[3+\frac{\left(1+\frac{4\alpha T}{3}\right)\left(1+\alpha T\right)+X}{\alpha T}\right]$$

**Figure 12:**
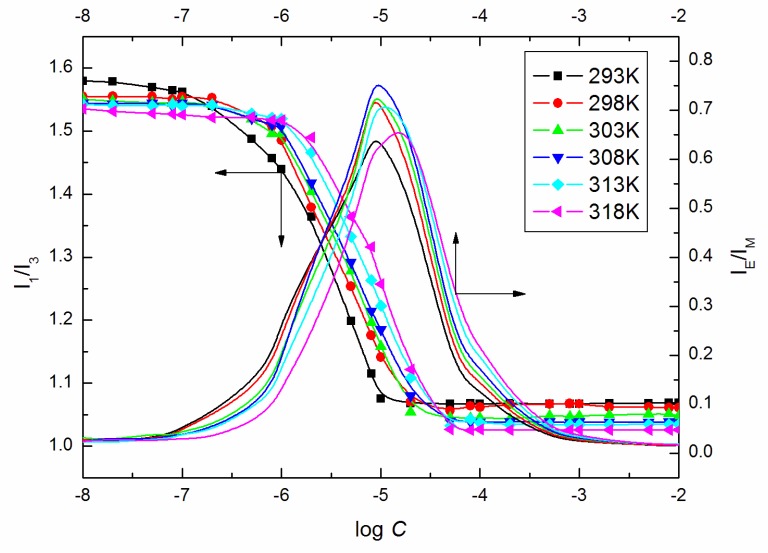
Plot of the values of $I_{E}/I_{M}$ and $I_{1}/I_{3}$ determined from the fluorescence spectra of pyrene in the aqueous solutions of ELP vs. log *C*.

Table S1 shows the variable character of $S_{0}$ and T which differs from the suggestions given by Sharma et al. [20,36]. They stated that $S_{0}$ is the constant in any liquid or solid-state system. However, fractional free volume (*f*) values, which are expressed in terms of the repulsive exponent of intermolecular potential, are characterized by the non-linear rise with T pointing out that the surfactant molecules mobility results in greater liquid (surfactant + water) disorder, which is due to irregular molecular packing [35]. Simultaneously the $C_{1}$, $X^{\prime }$, F, Γ and $\Gamma_{p}$ the decreasing values with the increasing temperature and concentration of the surfactant indicating the molecular ordering increase with the increasing values of T and *C* [37]. This is consistent with the conclusions drawn based on the viscosity measurements namely that the ELP molecules act as a structure maker at its high concentrations. One should bear in mind that ELP, as a nonionic surfactant possessing three amphiphilic chains, is a nonconventional surfactant that can be treated as a polymer. The coiling index was determined based on the pyrene fluorescence spectroscopy in order to reveal the changes of the chains’ conformation. It is the ratio of the excimer intensity $I_{E}$ (at ∼480 nm) and the monomer, $I_{M}=(I_{1}+I_{3})/2$, where $I_{1}$ and $I_{3}$ are the intensity of the first and the third vibrionic peaks in the pyrene emission spectra [38]. The high value of $I_{E}/I_{M}$ indicates the medium with a greater possibility of excimer formation and coiled surfactant chains. As can be seen in Figure 12, the highest coiling index values are found at the concentrations close to the ELP *CMC* values calculated from the surface tension measurements as well as the *C* values with the polarity index ($I_{1}/I_{3}$) characterized by a minimal value. Considering the concentration values higher than *CMC*, there is observed the $I_{E}/I_{M}$ values’ decrease with the increasing number of micelles providing more pyrene locations or micellar size or shape changes. At *C* higher than 10^−3^ M, $I_{E}/I_{M}$ is still constant with the lowest values indicating stretching of the surfactant chains, which is consistent with the f values which increase remarkably at *C* greater than 10^−3^ M at a given temperature (Table S1).

## 3. Materials and Methods

Kolliphor^®^ ELP (ELP) (Cremophor^®^ELP, Polyoxyl 35 Hydrogenated Castor Oil, Polyoxyl-35 Castor Oil) purchased from Sigma were used without further purification. Its aqueous solutions were obtained at the concentrations 10^−6^ to 10^−2^ M applying the doubly distilled and deionized water provided by a Destamat Bi18E distiller. The surface tension was measured by means of a Krüss K100C tensiometer according to the platinum ring tensiometer method (du Nouy’s method). The measurements of pure water surface tension at 293 K were aimed at the calibration of the tensiometer and glassware cleanliness control. There were performed ten successive measurements with the standard deviation not exceeding ±0.2 mN/m. The controlled temperature was within ±0.1 K. The measurements of surfactant aqueous solutions density were performed by means of a U-tube densitometer (DMA 5000 Anton Paar) at 293–318 K. The density and temperature measurement precision given by the producer was ±0.000005 g cm^−3^and ±0.001 K. The calculated uncertainty was 0.01%. The densitometer calibration with distilled and deionized water was regular.

The surfactant aqueous solutions viscosity was determined using the Anton Paar viscometer (AMVn) at 293–318 K ±0.01 K. Its precision was 0.0001 mPa s and the uncertainty was 0.3%. The steady-state fluorescence was measured at 293–318 K by means of a Hitachi F-2700 Fluorescence Spectrometer where pyrene was as a luminescence probe (*C* Py = 4 × 10^−6^ M). The fluorescence excitation for pyrene was induced at 335 nm and the range of the emission spectra recording was 350–600 nm at a scan speed of 300 nm/min. The widths of the excitation and emission slit widths were 5 nm.

## 4. Conclusions

In this paper, some physicochemical properties of the aqueous solutions of the nonionic surfactant, Kolliphor® ELP (ELP), are singled out, discussed and compared on the basis of the surface tension, density and viscosity measurements as well as fluorescence spectra. From the presented data and calculations, it is evident that ELP displays a greater propensity to be absorbed at the interface than form micelle in the bulk phase and that bond breaking predominates in the micellization and adsorption process. The ELP structure-making tendency at concentrations higher than 10^−3^ M was confirmed by the Hepler’s theory as well as the values of viscosity *B* coefficients and apparent molar volume. In addition, at this concentration range and a given temperature, the stretching of the surfactant chains were proved on the basis of pyrene emission spectra and the values of fractional free volume. On the other hand, at a concentration of ELP smaller than CMC, the polar head of the surfactant is strongly hydrated resulting in very compact conformation, probably because of water-bridging, which promotes gauche conformations.

## Figures and Tables

**Table 1:** Values of CMC, $C_{20}$, $CMC/C_{20}$, $\Pi_{CMC}$, $\Delta G_{mic}^{0}$, $\Delta S_{mic}^{0}$, $\Delta H_{mic}^{0}$, $\Gamma_{m}$, $A_{m}$, $\Delta G_{ad}^{0}$, $\Delta S_{ad}^{0}$ and $\Delta H_{ad}^{0}$ for the aqueous solutions of ELP at the temperatures from 293 to 318 K.

	T = 293K	298K	303K	308K	313K	318K
CMC (mol/dm^3^)	2.14 × 10^−5^	2.09 × 10^−5^	2.03 × 10^−5^	1.97 × 10^−5^	1.91 × 10^−5^	1.85 × 10^−5^
$C_{20}$ (mol/dm^3^)	3.83 × 10^−6^	3.82 × 10^−6^	3.79 × 10^−6^	3.74 × 10^−6^	3.65 × 10^−6^	3.53 × 10^−6^
$CMC/C_{20}$	5.59	5.47	5.35	5.26	5.23	5.24
$\Pi_{CMC}$ (mN/m)	32.91	32.82	32.72	32.59	32.43	32.29
$\Delta G_{mic}^{0}$ (kJ/mol)	−26.19	−26.70	−27.22	−27.75	-28.28	−28.81
$\Delta S_{mic}^{0}$ (kJ/mol K)	0.105					
$\Delta H_{mic}^{0}$ (kJ/mol)	4.58	4.59	4.59	4.59	4.59	4.58
$\Gamma_{m}$ (mol/m^2^)	3.17 × 10^−6^	3.11 × 10^−6^	3.06 × 10^−6^	3.01 × 10^-6^	2.95 × 10^-6^	2.90 × 10^-6^
$A_{m}$ (nm^2^)	0.524	0.533	0.542	0.552	0.563	0.572
$\Delta G_{ad}^{0}$ (kJ/mol)	−36.58	−37.23	−37.91	−38.58	−39.27	−39.93
$\Delta S_{ad}^{0}$ (kJ/mol K)	0.135					
$\Delta H_{ad}^{0}$ (kJ/mol)	2.98	3.00	3.00	3.00	2.99	3.00

## Supplementary Materials

The following are available online, Table S1: Values of $\tilde{V}$, $C_{1}$, $\tilde{\beta }$, X, $S_{0}$, F, $X^{\prime }$, Γ, f, $\Gamma_{p}$, K and $K^{\prime \prime }$ for the aqueous solutions of ELP at the temperatures from 293 to 318 K.
