# Supplementary material for: Temperature Effect on the Adsorption and Volumetric Properties of Aqueous Solutions of Kolliphor®ELP
Source: Molecules. 2020 Feb 9;25(3):743. doi: 10.3390/molecules25030743 (PMC7037185; doi:10.3390/molecules25030743)
Supplement: Supplementary file 1 [file molecules-25-00743-s001.pdf]

SUPPLEMENTARY MATERIALS

Temperature Effect on the Adsorption and Volumetric Properties of Aqueous Solutions of Kolliphor®ELP

Katarzyna Szymczyk*, Magdalena Szaniawska and Joanna Krawczyk

Department of Interfacial Phenomena, Institute of Chemical Sciences, Faculty of Chemistry, Maria Curie-Skłodowska University in Lublin, Maria Curie-Skłodowska Sq. 3, 20-031 Lublin, Poland
Phone：(48-81) 537-5538
Fax：(48-81) 533-3348
*Correspondence：katarzyna.szymczyk@poczta.umcs.lublin.pl (K. Szymczyk)

Table S1. Values of $\tilde{V}$, C_1, $\tilde{\beta}$, X, S_0, F, X', Γ, f, Γ_p, K and K'' for the aqueous solutions of ELP at the temperatures from 293 to 318 K.

	C	293K	298K	303K	308K	313K	318K
$\tilde{V}$	10^{-5}	1.0681	1.0848	1.1026	1.1215	1.1416	1.1629
	$2x10^{-5}$	1.0681	1.0848	1.1026	1.1215	1.1416	1.1629
	$5x10^{-5}$	1.0681	1.0848	1.1026	1.1215	1.1416	1.1630
	$8x10^{-5}$	1.0681	1.0848	1.1026	1.1215	1.1416	1.1630
	10^{-4}	1.0682	1.0848	1.1027	1.1215	1.1417	1.1630
	$2x10^{-4}$	1.0683	1.0850	1.1028	1.1216	1.1418	1.1631
	$5x10^{-4}$	1.0685	1.0852	1.1030	1.1218	1.1419	1.1632
	$8x10^{-4}$	1.0687	1.0854	1.1032	1.1221	1.1422	1.1634
	10^{-3}	1.0690	1.0856	1.1034	1.1223	1.1423	1.1635
	$2x10^{-3}$	1.0698	1.0865	1.1042	1.1230	1.1430	1.1642
	$5x10^{-3}$	1.0723	1.0889	1.1067	1.1255	1.1455	1.1667
	$8x10^{-3}$	1.0745	1.0912	1.1089	1.1278	1.1478	1.1690
	10^{-2}	1.0764	1.0929	1.1105	1.1291	1.1488	1.1698
C_1	10^{-5}	20.3750	17.4834	15.4432	13.9331	12.7715	11.8545
	$2x10^{-5}$	20.3730	17.4822	15.4421	13.9329	12.7711	11.8543
	$5x10^{-5}$	20.3710	17.4809	15.4406	13.9327	12.7706	11.8538
	$8x10^{-5}$	20.3657	17.4792	15.4399	13.9325	12.7702	11.8534
	10^{-4}	20.3583	17.4786	15.4364	13.9321	12.7688	11.8529
	$2x10^{-4}$	20.3303	17.4569	15.4230	13.9275	12.7625	11.8501
	$5x10^{-4}$	20.2835	17.4284	15.4094	13.9107	12.7575	11.8454
	$8x10^{-4}$	20.2410	17.4045	15.3876	13.8912	12.7446	11.8369
	10^{-3}	20.1879	17.3682	15.3695	13.8833	12.7383	11.8318
	$2x10^{-3}$	20.0034	17.2532	15.2946	13.8331	12.7040	11.8083
	$5x10^{-3}$	19.5278	16.9378	15.0743	13.6735	12.5852	11.7178
	$8x10^{-3}$	19.1156	16.6615	14.8801	13.5320	12.4793	11.6371
	10^{-2}	18.7756	16.4544	14.7511	13.4517	12.4308	11.6098
$\tilde{\beta}$	10^{-5}	3.8265	4.1502	4.5198	4.9423	5.4277	5.9861
	$2x10^{-5}$	3.8267	4.1504	4.5200	4.9423	5.4279	5.9862
	$5x10^{-5}$	3.8268	4.1505	4.5204	4.9424	5.4282	5.9866
	$8x10^{-5}$	3.8273	4.1508	4.5205	4.9425	5.4284	5.9869
	10^{-4}	3.8279	4.1509	4.5213	4.9426	5.4291	5.9873
	$2x10^{-4}$	3.8304	4.1540	4.5244	4.9442	5.4323	5.9893
	$5x10^{-4}$	3.8345	4.1582	4.5275	4.9500	5.4349	5.9928
	$8x10^{-4}$	3.8383	4.1617	4.5325	4.9568	5.4414	5.9990
	10^{-3}	3.8430	4.1670	4.5367	4.9595	5.4446	6.0028
	$2x10^{-3}$	3.8597	4.1842	4.5542	4.9773	5.4624	6.0202
	$5x10^{-3}$	3.9052	4.2335	4.6077	5.0353	5.5255	6.0891
	$8x10^{-3}$	3.9477	4.2794	4.6574	5.0893	5.5842	6.1529
	10^{-2}	3.9850	4.3157	4.6919	5.1211	5.6120	6.1750
X	10^{-5}	-0.5882	-0.5558	-0.5230	-0.4902	-0.4575	-0.4251
	$2x10^{-5}$	-0.5882	-0.5558	-0.5230	-0.4902	-0.4575	-0.4251

	5x10^{-5}	-0.5881	-0.5557	-0.5230	-0.4902	-0.4575	-0.4251
	8x10^{-5}	-0.5881	-0.5557	-0.5230	-0.4902	-0.4575	-0.4251
	10^{-4}	-0.5880	-0.5557	-0.5229	-0.4902	-0.4574	-0.4251
	2x10^{-4}	-0.5878	-0.5554	-0.5227	-0.4901	-0.4572	-0.4250
	5x10^{-4}	-0.5873	-0.5550	-0.5224	-0.4897	-0.4571	-0.4248
	8x10^{-4}	-0.5869	-0.5547	-0.5220	-0.4892	-0.4567	-0.4245
	10^{-3}	-0.5864	-0.5542	-0.5216	-0.4890	-0.4565	-0.4243
	2x10^{-3}	-0.5847	-0.5526	-0.5202	-0.4877	-0.4554	-0.4233
	5x10^{-3}	-0.5799	-0.5480	-0.5158	-0.4836	-0.4515	-0.4197
	8x10^{-3}	-0.5756	-0.5439	-0.5119	-0.4798	-0.4479	-0.4164
	10^{-2}	-0.5718	-0.5406	-0.5091	-0.4776	-0.4463	-0.4153
	10^{-5}	0.955981	0.918851	0.879754	0.839011	0.796822	0.753574
	2x10^{-5}	0.95596	0.918832	0.879727	0.839005	0.796807	0.753567
	5x10^{-5}	0.95594	0.918811	0.879693	0.838999	0.796784	0.75354
	8x10^{-5}	0.955884	0.918784	0.879679	0.838993	0.796768	0.753519
	10^{-4}	0.955808	0.918773	0.879597	0.838979	0.796708	0.753491
	2x10^{-4}	0.955518	0.918427	0.879288	0.838834	0.796447	0.753344
S_0	5x10^{-4}	0.95503	0.91797	0.878974	0.8383	0.796235	0.753088
	8x10^{-4}	0.954583	0.917585	0.878467	0.83768	0.795695	0.752637
	10^{-3}	0.954023	0.916997	0.878046	0.837429	0.795432	0.752362
	2x10^{-3}	0.952041	0.91511	0.876283	0.835813	0.793983	0.751097
	5x10^{-3}	0.946694	0.909743	0.870947	0.830561	0.788862	0.746152
	8x10^{-3}	0.941754	0.904795	0.866039	0.825739	0.784168	0.74163
	10^{-2}	0.937447	0.900923	0.862671	0.822931	0.78197	0.740074
	10^{-5}	1.5046	1.4536	1.4017	1.3493	1.2964	1.2435
	2x10^{-5}	1.5046	1.4536	1.4017	1.3493	1.2964	1.2435
	5x10^{-5}	1.5046	1.4535	1.4016	1.3493	1.2964	1.2434
	8x10^{-5}	1.5045	1.4535	1.4016	1.3493	1.2964	1.2434
	10^{-4}	1.5044	1.4535	1.4015	1.3492	1.2963	1.2434
	2x10^{-4}	1.5040	1.4530	1.4011	1.3490	1.2960	1.2432
F	5x10^{-4}	1.5033	1.4524	1.4007	1.3484	1.2957	1.2429
	8x10^{-4}	1.5027	1.4519	1.4000	1.3476	1.2950	1.2423
	10^{-3}	1.5019	1.4511	1.3995	1.3473	1.2947	1.2420
	2x10^{-3}	1.4991	1.4485	1.3972	1.3452	1.2929	1.2405
	5x10^{-3}	1.4917	1.4413	1.3902	1.3386	1.2866	1.2345
	8x10^{-3}	1.4848	1.4347	1.3839	1.3325	1.2808	1.2290
	10^{-2}	1.4789	1.4296	1.3795	1.3290	1.2781	1.2272
	10^{-5}	-1.1253	-1.1533	-1.1820	-1.2114	-1.2416	-1.2725
	2x10^{-5}	-1.1253	-1.1533	-1.1820	-1.2114	-1.2416	-1.2725
	5x10^{-5}	-1.1254	-1.1533	-1.1821	-1.2115	-1.2417	-1.2725
	8x10^{-5}	-1.1254	-1.1533	-1.1821	-1.2115	-1.2417	-1.2725
	10^{-4}	-1.1255	-1.1533	-1.1821	-1.2115	-1.2417	-1.2726
	2x10^{-4}	-1.1257	-1.1536	-1.1823	-1.2116	-1.2419	-1.2727
X'	5x10^{-4}	-1.1261	-1.1539	-1.1826	-1.2120	-1.2420	-1.2728
	8x10^{-4}	-1.1264	-1.1542	-1.1829	-1.2124	-1.2424	-1.2732
	10^{-3}	-1.1268	-1.1547	-1.1833	-1.2126	-1.2426	-1.2734
	2x10^{-3}	-1.1283	-1.1561	-1.1845	-1.2137	-1.2437	-1.2743
	5x10^{-3}	-1.1324	-1.1600	-1.1884	-1.2175	-1.2473	-1.2778
	8x10^{-3}	-1.1361	-1.1637	-1.1920	-1.2210	-1.2507	-1.2810
	10^{-2}	-1.1394	-1.1665	-1.1944	-1.2230	-1.2522	-1.2822
	10^{-5}	5.9944	5.6159	5.3479	5.1480	4.9923	4.8671
	2x10^{-5}	5.9941	5.6157	5.3477	5.1480	4.9923	4.8670
	5x10^{-5}	5.9939	5.6155	5.3475	5.1480	4.9922	4.8670
	8x10^{-5}	5.9932	5.6153	5.3475	5.1480	4.9921	4.8669
	10^{-4}	5.9922	5.6152	5.3470	5.1479	4.9919	4.8668
	2x10^{-4}	5.9886	5.6124	5.3452	5.1473	4.9911	4.8665
Γ	5x10^{-4}	5.9824	5.6087	5.3434	5.1450	4.9904	4.8658
	8x10^{-4}	5.9769	5.6055	5.3406	5.1424	4.9887	4.8646
	10^{-3}	5.9699	5.6008	5.3382	5.1414	4.9878	4.8639
	2x10^{-3}	5.9458	5.5857	5.3283	5.1347	4.9832	4.8607
	5x10^{-3}	5.8836	5.5443	5.2992	5.1134	4.9671	4.8481
	8x10^{-3}	5.8296	5.5081	5.2736	5.0945	4.9527	4.8369
	10^{-2}	5.7851	5.4809	5.2565	5.0838	4.9461	4.8331
f	10^{-5}	0.142971	0.151152	0.157533	0.162654	0.166881	0.170443

	$2x10^{-5}$	0.142977	0.151155	0.157536	0.162654	0.166882	0.170444
	$5x10^{-5}$	0.142982	0.151159	0.157541	0.162655	0.166884	0.170446
	$8x10^{-5}$	0.142996	0.151164	0.157543	0.162656	0.166886	0.170447
	10^{-4}	0.143016	0.151166	0.157555	0.162657	0.166891	0.17045
	$2x10^{-4}$	0.143091	0.151231	0.157599	0.162673	0.166914	0.170461
	$5x10^{-4}$	0.143217	0.151317	0.157643	0.162733	0.166934	0.17048
	$8x10^{-4}$	0.143331	0.151388	0.157715	0.162802	0.166982	0.170514
	10^{-3}	0.143474	0.151498	0.157774	0.162829	0.167006	0.170534
	$2x10^{-3}$	0.143972	0.151844	0.15802	0.163007	0.167135	0.170629
	$5x10^{-3}$	0.145274	0.152804	0.158749	0.163574	0.167587	0.170995
	$8x10^{-3}$	0.146421	0.153655	0.159398	0.164082	0.167991	0.171323
	10^{-2}	0.147382	0.1543	0.159833	0.164371	0.168178	0.171435
	10^{-5}	10.0209	8.5751	7.5549	6.7999	6.2191	5.7606
	$2x10^{-5}$	10.0198	8.5744	7.5544	6.7998	6.2189	5.7605
	$5x10^{-5}$	10.0188	8.5738	7.5536	6.7997	6.2186	5.7603
	$8x10^{-5}$	10.0162	8.5729	7.5533	6.7996	6.2184	5.7600
	10^{-4}	10.0125	8.5726	7.5515	6.7994	6.2177	5.7598
	$2x10^{-4}$	9.9985	8.5618	7.5448	6.7971	6.2146	5.7584
Γ_p	$5x10^{-4}$	9.9751	8.5475	7.5381	6.7887	6.2121	5.7560
	$8x10^{-4}$	9.9538	8.5356	7.5271	6.7789	6.2056	5.7518
	10^{-3}	9.9273	8.5174	7.5181	6.7750	6.2025	5.7492
	$2x10^{-3}$	9.8350	8.4599	7.4806	6.7499	6.1853	5.7375
	$5x10^{-3}$	9.5973	8.3022	7.3705	6.6701	6.1259	5.6923
	$8x10^{-3}$	9.3911	8.1641	7.2734	6.5993	6.0730	5.6519
	10^{-2}	9.2211	8.0605	7.2089	6.5592	6.0487	5.6382
	10^{-5}	9.6875	8.2417	7.2216	6.4666	5.8858	5.4272
	$2x10^{-5}$	9.6865	8.2411	7.2210	6.4665	5.8856	5.4272
	$5x10^{-5}$	9.6855	8.2405	7.2203	6.4664	5.8853	5.4269
	$8x10^{-5}$	9.6828	8.2396	7.2200	6.4663	5.8851	5.4267
	10^{-4}	9.6791	8.2393	7.2182	6.4660	5.8844	5.4265
	$2x10^{-4}$	9.6652	8.2285	7.2115	6.4638	5.8813	5.4251
K	$5x10^{-4}$	9.6418	8.2142	7.2047	6.4553	5.8787	5.4227
	$8x10^{-4}$	9.6205	8.2023	7.1938	6.4456	5.8723	5.4185
	10^{-3}	9.5940	8.1841	7.1848	6.4417	5.8692	5.4159
	$2x10^{-3}$	9.5017	8.1266	7.1473	6.4165	5.8520	5.4041
	$5x10^{-3}$	9.2639	7.9689	7.0372	6.3367	5.7926	5.3589
	$8x10^{-3}$	9.0578	7.8308	6.9400	6.2660	5.7397	5.3186
	10^{-2}	8.8878	7.7272	6.8756	6.2259	5.7154	5.3049
	10^{-5}	-3.6931	-2.6259	-1.8737	-1.3185	-0.8934	-0.5602
	$2x10^{-5}$	-3.6924	-2.6254	-1.8733	-1.3185	-0.8933	-0.5601
	$5x10^{-5}$	-3.6916	-2.6249	-1.8727	-1.3184	-0.8931	-0.5600
	$8x10^{-5}$	-3.6897	-2.6243	-1.8725	-1.3183	-0.8930	-0.5598
	10^{-4}	-3.6869	-2.6240	-1.8712	-1.3182	-0.8924	-0.5596
	$2x10^{-4}$	-3.6766	-2.6161	-1.8663	-1.3165	-0.8902	-0.5586
K'	$5x10^{-4}$	-3.6593	-2.6055	-1.8613	-1.3103	-0.8883	-0.5569
	$8x10^{-4}$	-3.6436	-2.5967	-1.8532	-1.3031	-0.8836	-0.5538
	10^{-3}	-3.6240	-2.5833	-1.8466	-1.3003	-0.8813	-0.5520
	$2x10^{-3}$	-3.5559	-2.5409	-1.8190	-1.2818	-0.8688	-0.5435
	$5x10^{-3}$	-3.3804	-2.4246	-1.7379	-1.2233	-0.8255	-0.5108
	$8x10^{-3}$	-3.2282	-2.3227	-1.6664	-1.1715	-0.7870	-0.4817
	10^{-2}	-3.1027	-2.2463	-1.6190	-1.1421	-0.7693	-0.4718
